# Elucidating Sulfide Activation Mode in Metal-Catalyzed Sulfoxidation Reactivity

**DOI:** 10.1021/acs.inorgchem.2c00037

**Published:** 2022-02-28

**Authors:** Diego Garay-Ruiz, Cristiano Zonta, Silvia Lovat, Joan González-Fabra, Carles Bo, Giulia Licini

**Affiliations:** † Barcelona Institute of Science & Technology (BIST), Institute of Chemical Research of Catalonia (ICIQ), Av. Països Catalans, 16, 43007 Tarragona, Spain; ‡ Departament de Química Física i Inorgànica, Universitat Rovira i Virgili (URV), C/Marcel·lí Domingo s/n, 43007 Tarragona, Spain; § Dipartimento di Scienze Chimiche, Università degli Studi di Padova and CIRCC, Padova Unit, via Marzolo 1, 35131 Padova, Italy

## Abstract

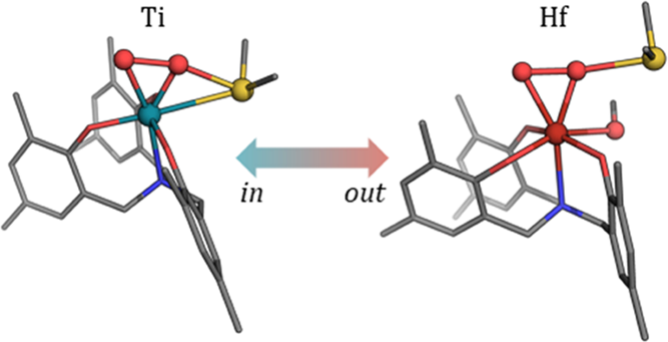

Interest in the catalytic activation of peroxides, together with the requirement of stereoselectivity for the production of enantiopure sulfoxides, has made sulfoxidation the ideal playground for theoretical and experimental physical organic chemists investigating oxidation reactivity. Efforts have been dedicated for elucidating the catalytic pathway regarding these species and for dissecting out the dominant factors influencing the yield and stereochemistry. In this article, Ti(IV) and Hf(IV) aminotriphenolate complexes have been prepared and investigated as catalysts in the presence of peroxides in sulfide oxidation. Experimental results have been combined with theoretical calculations obtaining detailed mechanistic information on oxygen transfer processes. The study revealed that steric issues are mainly responsible for the formation of intermediates in the oxidation pathway. In particular, we could highlight the occurrence of a blended situation where the steric effects of sulfides, ligands, and oxidants influence the formation of different intermediates and reaction pathways.

## Abstract 2

Steric issues are mainly responsible for the formation of intermediates in the catalytic activation of the peroxide pathway. In particular, we could highlight the occurrence of a blended situation where the steric effects of sulfides, ligands, and oxidants influence the formation of different intermediates and reaction pathways.

## Introduction

**Scheme 1:**
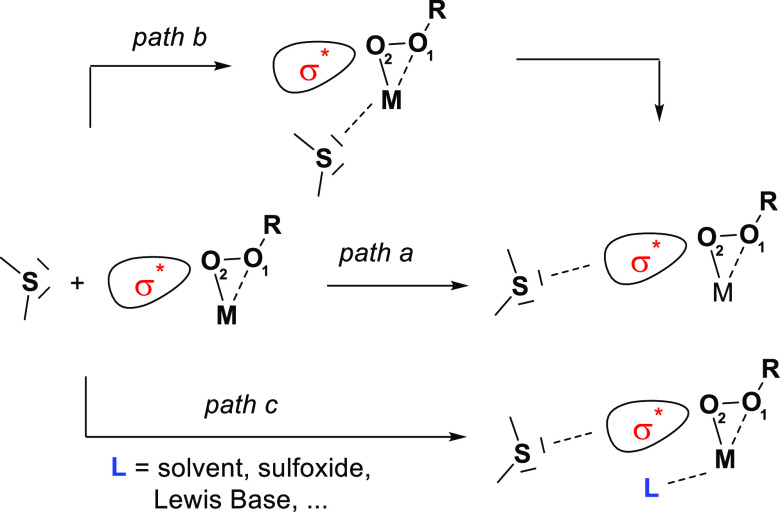
Possible Sulfide Attack Modes, with (Path b) and without (Path a) Thioether Precoordination to the Metal Center An external ligand (e.g., Lewis base, solvent, or sulfoxide) can also bind to the metal center.

The reactivity of peroxides bound to a metal ion is remarkably diverse and the factors dictating the particular mode of reactivity are continuously debated.^1−6^ Homolytic and heterolytic O–O bond cleavages have been observed in a variety of complexes and, among the different factors that can influence the reactivity, interactions related to the substrate–catalyst recognition event have attracted increasing interest being central in the final outcome, in particular for stereoselective processes.^7−16^ Even if the mechanism of oxygen transfer continues to be a controversial subject, widely accepted is that a “Sharpless type” pathway is involved in the oxidation step (Scheme 1, path a) and the active species is an η^2^ coordinated alkyl (or hydro) peroxo complex. The nucleophilic attack of the substrate to the electrophilic peroxo oxygen follows the antibonding σ* orbital (O_1_–O_2_). While theoretical studies on sulfide oxidation have been mainly directed to the explanation of the different stereoselective outcomes, the discussion remains controversial on the existence of a previous intermediate where the sulfide (or sulfoxide) is coordinated to the metal center prior to oxidation (Scheme 1, path b). In addition, in recent years, we reported several examples in which the presence of extra-ligands able to coordinate to the metal center (Scheme 1, path c) can have noteworthy consequences on the reactivity of the metal peroxide.^2,17^

In the present paper, experimental and theoretical studies on the oxidation of methyl-*p*-tolyl sulfide using two different metal complexes, namely, Ti(IV) and Hf(IV), have been combined to obtain detailed information on the thioether catalytic cycle. These two metals have been chosen because they offer the same coordination geometry but different atomic sizes. The study highlights a mechanistic pathway in which a blended situation among the three possible pathways takes place, modulated by steric and electronic factors. In particular, while in the presence of steric hindrance, the sulfide prefers to avoid metal coordination, the preferred mechanism in the absence of steric impediments involves binding of the sulfide to the metal center.

## Experimental Section

### General Considerations

NMR spectra were recorded at 301 K on Bruker 400 Avance III BBi-z grad 5 mm and Bruker Avance III 500MHz instruments. All of the ^1^H NMR spectra were referenced to the residual isotopic impurity of the solvent. Synthesis of the ligand and **Ti** complex have been reported previously and **Hf** is described in the Supporting Information.

### Computational Details

All calculations were carried out with the PBE-D3 functionals (Perdew–Burke–Ernzerhof correlation/exchange^18,19^ and Grimme’s D3 dispersion^20^) and a TZ2P basis set, including ZORA^21−23^ relativistic corrections and the COSMO^24^ solvent model for chloroform, in ADF 2016.^25,26^ The reported Gibbs free energies (except where otherwise stated) include Martin^27^ entropic corrections at the working temperature (298.15 K) and considering the density of chloroform at this temperature (1.49 g/mL). All structures were optimized without any geometric constraints.

All minima and transition states were confirmed by harmonic vibrational analysis, identifying 0 or 1 imaginary frequencies, respectively. Connectivity between minima and TSs was checked by displacing the reactive eigenmode of the transition states.

Fragment analysis was also performed in ADF 2016, selecting two disjoint regions for the sulfide substrate and the rest of the metal complex. NCI and DORI analyses for noncovalent interactions were done with a *medium* grid.

## Results and Discussion

### Synthesis of the Complexes and Catalytic activity

**Scheme 2:**
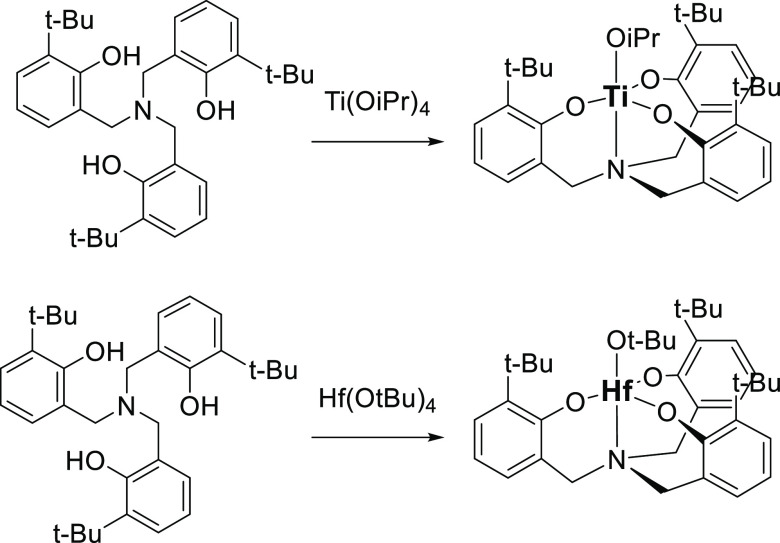
Ti (**Ti**^**tBu**^) and Hf (**Hf**^**tBu**^) Aminotriphenolate Synthesis

Aminotriphenolate Ti(IV) and Hf(IV) complexes have been prepared from ortho-t-butyl triphenolamine^28^ with the corresponding tetraalkoxy metal precursors Scheme 2 (see Section 2 in the Supporting Information).^29−31^

While previous studies on the catalytic activity toward sulfoxidation of **Ti** have shown that this catalyst performs well in terms of reactivity using alkyl peroxide or hydrogen peroxide as the terminal oxidant,^30^**Hf** reactivity was completely novel. For this reason, we decided to apply standard reaction conditions for both catalysts with 0.1 M concentration of methyl-*p*-tolyl sulfide **S** and of the peroxide, and 0.001 M concentration of the catalysts (1%), under the same reaction conditions. The results of the catalytic tests are shown in Table 1, and this comprehends the yield of the reaction, the final time, and the final sulfoxide (**SO**) and sulfone (**SO**_**2**_) ratio. Initial rates were also calculated for the reactions for the first and second oxidation steps. All of the complexes own the capability to transfer oxygen from cumyl hydroperoxide **CHP** to the thioether **S**. From the experimental data, it has been possible to extrapolate kinetic constants for the first oxidation process, sulfide to sulfoxide oxidation (*k*_1_), and for the second oxidation process, sulfoxide to sulfone (*k*_2_, Table 1). This has been calculated by nonlinear fitting of a first-order equation of the initial 20% of the reaction. This is to have the minimal influence of the degradation of the complex and/or of the oxidant, and to avoid the effect, already shown by our previous work, which can have the coordination of the newly formed sulfoxide to the reactivity of the metal complex. However, even though the same ligand is present for the two different metals, experimental kinetic values are not straightforward for interpretation.

**Table 1:**
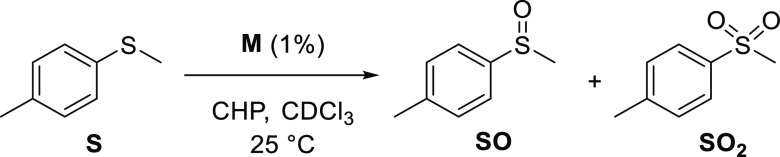
Oxidation of *p*-Tolyl Methyl Sulfide S Using CHP^a^

metal	conversion^b^ (%)	time (h)	**SO:SO**_**2**_^b^	*k*_1_^c^ (mol^–3^ h^–1^)	*k*_2_^c^ (mol^–3^ h^–1^)	*k*_1_/*k*_2_
**Ti**	99	40	80:20	1583	380	4.17
**Hf**	99	3	30:70	8030	31 200	0.26

a Reaction conditions: 25 °C, [**S**]_0_ = [**CHP**]_0_ = 0.1 M, and [**M**] = 0.001 M.

b Sulfide conversion determined by ^1^H NMR analysis on the crude reaction mixture after complete oxidant consumption (iodometric test).

c Determined using nonlinear fitting of a first-order equation of the first 20% of the reaction.

### Theoretical Calculations

**Figure 1:**
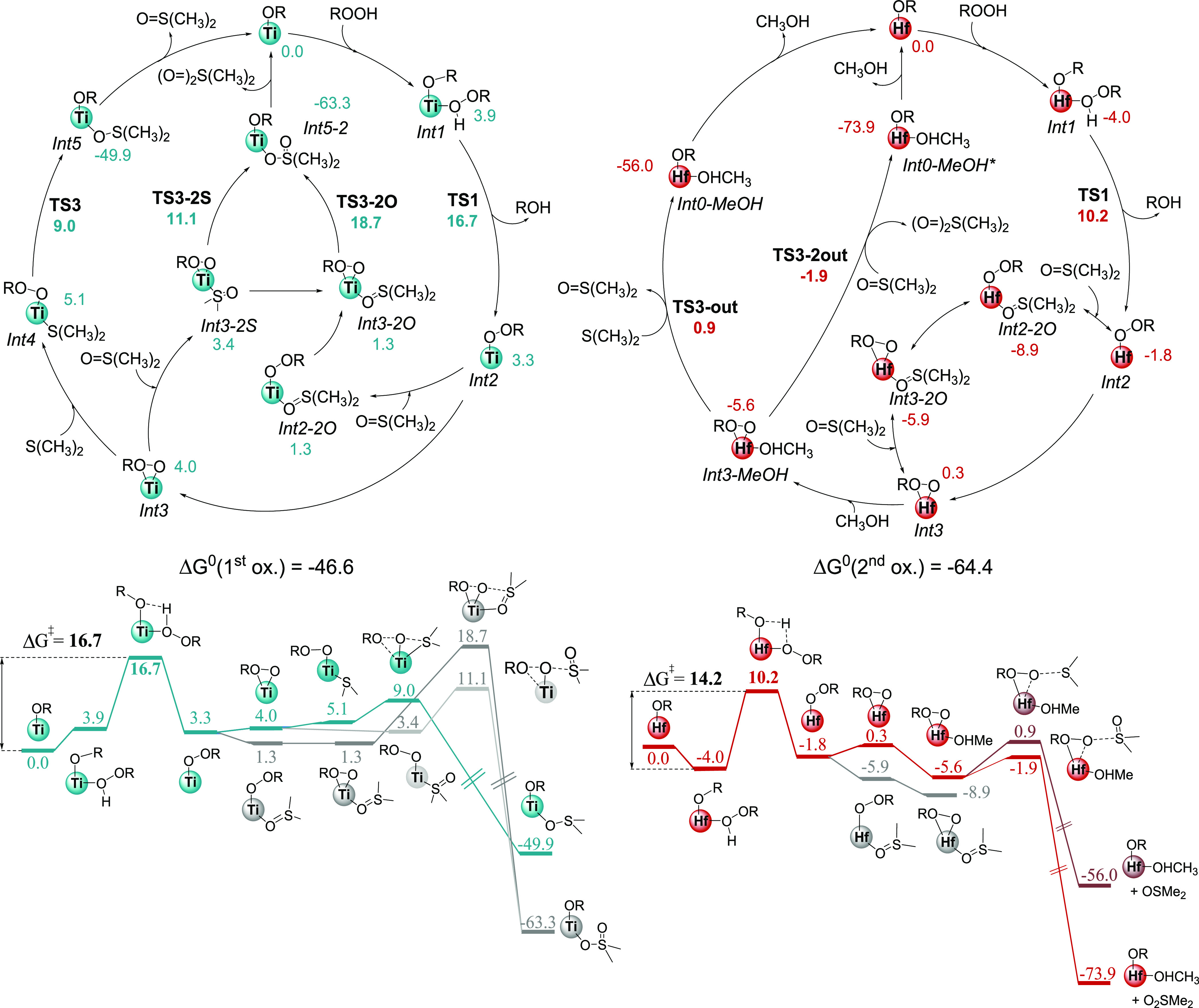
Mechanistic proposals for M = Ti (blue) and M = Hf (red), as catalytic cycles (above) and energy profiles (below). Gibbs free energies, in kcal·mol^–1^, including Martin free energy corrections for chloroform as the solvent at 298.15 K.

**Scheme 3:**
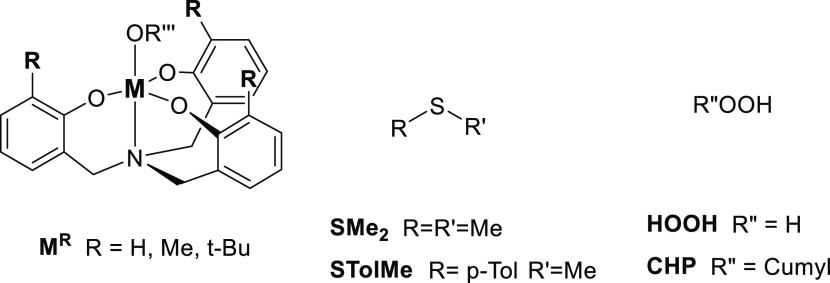
Model Compounds Used in the Computational Study

Experimental results show a key difference in the product selectivity of **Ti** and **Hf**-aminotriphenolate catalysts: under the same reaction conditions, **Ti** favors the sulfoxide formation (**SO**) while **Hf** mainly affords sulfone (**SO**_**2**_). Additionally, catalysis with **Hf** is much faster than with **Ti**. To explain the selectivity and kinetics of the reactions, we investigated the corresponding reaction mechanisms through DFT calculations for the two metals, considering the formation of the previously mentioned η^2^ peroxo intermediate as the active species for the oxygen transfer process. To limit the system size and make calculations more affordable, we considered **SMe**_**2**_ as the substrate model and a methyl-substituted aminotriphenolate ligand **M**^**Me**^ instead of the experimental *p*-tolyl sulfide **STolMe** and *tert*-butyl-substituted ligand **M**^**t-Bu**^ (Scheme 3). The corresponding catalytic cycles for **Ti** and **Hf** are depicted in Figure 1.

The rate-determining step of the reaction is **TS1**, corresponding to the formation of the η^1^-alkylperoxide complex **Int2** with the release of cumyl alcohol. The corresponding free energy barriers are 16.7 kcal/mol for **Ti** and 14.2 kcal/mol for **Hf**, justifying the better catalytic performance of the latter complex. From there on, there are important differences in the reactivity of the two metals. **Ti** prefers to bind to the sulfur-bearing substrate first (inner-sphere-like, *path b* in Scheme 1), while for **Hf** the lowest-energy routes involve the coordination of an additional ligand (namely, methanol) to the metal. Then, the substrate approaches without binding: outer-sphere-like or *path c*.

The difference in cation sizes (with **Hf** ≫ **Ti**) is the main factor explaining the differences in reactivity: **Hf** has a larger tendency to add additional ligands on its equatorial plane, forming a more stable species. In contrast, the equivalent species with **Ti** are higher in energy because the smaller metal has more difficulties expanding its coordination sphere. To explain the selectivity of the reaction, we must take into account that for the second oxidation to proceed, some sulfoxide must have already been produced from the first oxidation step. Consequently, when the sulfoxide **SO** has already been oxidized and depleted, the process will be halted. Moreover, sulfoxide binding to the metal allows the formation of quite stable complexes for **Hf** (**Int2-2O**, at −8.9 kcal/mol), entering in competition with the equatorial alcohol that controls the outer-sphere-like mechanism. Also, the highest-lying transition state for both metals is **TS1**, which does not lead to any speciation. However, if we look at the lowest-energy channels appearing after **TS1**, we have that the preferred S–O bond formation transition states are **TS3** for **Ti** (inner-sphere-like, leading to sulfoxide) and **TS3-2out** for **Hf** (outer-sphere-like, leading to sulfone).

Therefore, kinetic constants for sulfoxide and sulfone formation (*k*_1_ and *k*_2_) cannot be directly compared with the individual barriers: the product selection steps **Ti-TS3** and **Hf-TS3-2out** are different from the common rate-determining step **M-TS1**, with the expected product channels agreeing with the experimental selectivities: **Ti** to sulfoxide and **Hf** to sulfone.

Focusing on substrate activation, the previous studies^2^ already demonstrated that the trigonal bipyramid hydroperoxo complex analogous to **Int3** could not directly form the S–O bond (which would correspond to *path a* in Scheme 1), due to orbital shielding. Therefore, it is necessary to extend the coordination sphere of titanium to an octahedral-like geometry for the reaction to proceed. In the inner-sphere mechanisms, the substrate already plays this role but the outer-sphere paths require an additional equatorial ligand. Alcohols are common additives for this kind of reaction setup that can bind to the metal center and fulfill this purpose, as explored in the previously mentioned study. Following this approach, we considered methanol as the auxiliary ligand in our mechanistic characterization.

The outer-sphere alternative pathway is strongly disfavored for **Ti**, with the corresponding methanol-activated outer-**TS3** equivalent lying almost 9 kcal/mol higher (17.9 kcal/mol) than the inner-like **TS3**. On the other side, both possible pathways are quite close in energy for **Hf**, either for the first or for the second oxidation routes: see Table S2 in the Supporting Information for further information.

Nevertheless, additional questions about the true inner-sphere nature of **Int4** and **TS3** can be raised upon careful inspection of the corresponding structures. Although the metal center is undeniably octahedral-like, the sulfur–metal distances are quite long: 2.81 Å in **Ti-Int4** and 3.22 Å in **Ti-TS3**. Moreover, Bader analysis^32^ does not show any covalent Ti–S contribution in the transition state, while there is a bond critical point (BCP) indicating covalent bonding in **Int4**, despite the obvious influence of the sulfide in the coordination environment of the metal.

To clarify these aspects, we characterized several variations of **TS3** with some structural modifications on the peroxide and the alkyl substituents in the aminotriphenolate scaffold. From the original structures (**CumOOH, M**^**Me**^), we devised a simpler model in which the cumyl hydroperoxide was substituted by hydrogen peroxide (**HOOH, M**^**Me**^) and a more complex one including *t*-butyl groups in the ligand **{CumOOH, M**^**tBu**^**}**. For the three types of structures, both the inner-sphere and the outer-sphere (bearing MeOH) transition states were computed, for a total of six different TS per metal.

**Figure 2:**
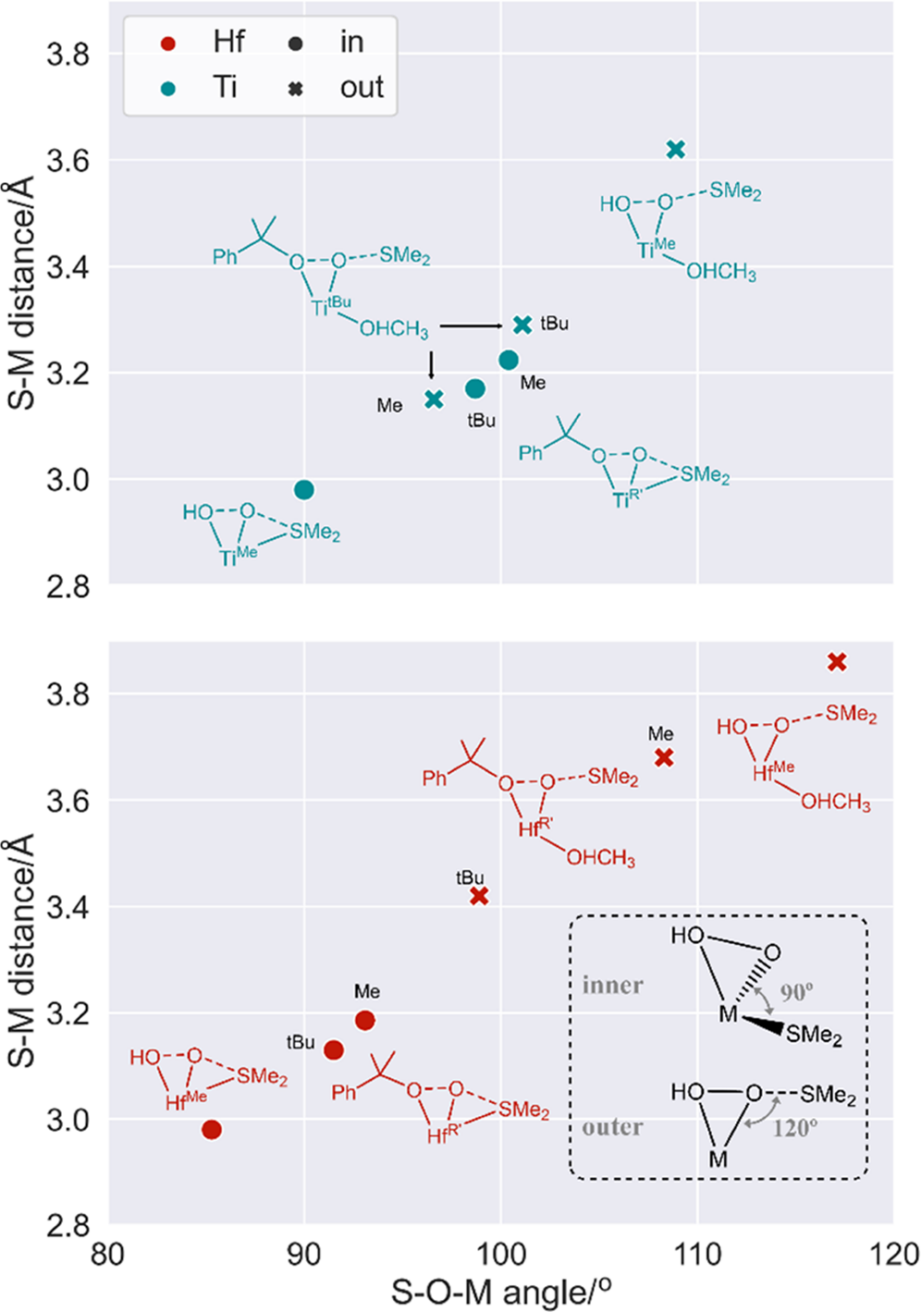
Mapping of the S–M distance and S–O–M angle for the series of 12 sulfoxidation transition states. Above the graph, ideal structures with the corresponding S–O–M angle values for reference.

We extracted key geometric parameters from the 12 transition state structures and built the geometry map shown in Figure 2, which illustrates the complexity of the inner/outer-sphere labeling in this kind of systems. It is only in the simplest case (**HOOH, M**^**Me**^), where we have a clearer inner/outer distinction for the two metals, with angle values closer to the “ideal” model limit situations. When the structures get more complex, as in (**CumOOH, M**^**Me**^) and (**CumOOH, M**^**tBu**^), the distinction becomes fuzzier, and we encounter a continuum of angle and distance values that do not fully match with the expected categories.

If we look at the transition states that we have labeled the outer sphere when the substrate includes the cumyl group, we find that their structures differ remarkably from the pure-outer-sphere reference found for the **{HOOH, M**^**Me**^**}** case. The hapticity of the peroxo group changes between η^1^ and η^2^ modes depending on the bulk of the substituent. When there is more steric congestion, a more η^1^-like mode is favored, allowing the sulfide to be closer to the metal. Regarding this diversity, we will refer to these attack modes as “in” and “out” modes instead of inner-sphere and outer-sphere along the discussion. Herein, the tags refer to the moiety that makes the coordination environment of the metal octahedral-like, which might be either sulfide (“in”) or methanol (“out”).

As shown in Table 2, the “in” transition states are clearly favored for titanium, being between 6 and 9 kcal/mol more stable than the “out” equivalents. In contrast, both modes are much alike in the case of hafnium, whose larger size allows for more stable binding of the additional methanol molecule present in the “out” structures.

**Table 2:** Differences between Gibbs Free Energies, in kcal/mol, between the “In” and “Out” Sulfoxidation Transition States for **Ti** and **Hf**

**systems**	***G*(TS**_**out**_**)** – ***G*(TS**_**in**_**)** (kcal·mol^–1^)
**HOOH, Ti**^**Me**^	6.5
**CumOOH, Ti**^**Me**^	9.0
**CumOOH, Ti**^**tBu**^	6.2
**HOOH, Hf**^**Me**^	–1.2
**CumOOH, Hf**^**Me**^	–2.1
**CumOOH, Hf**^**tBu**^	1.3

**Figure 3:**
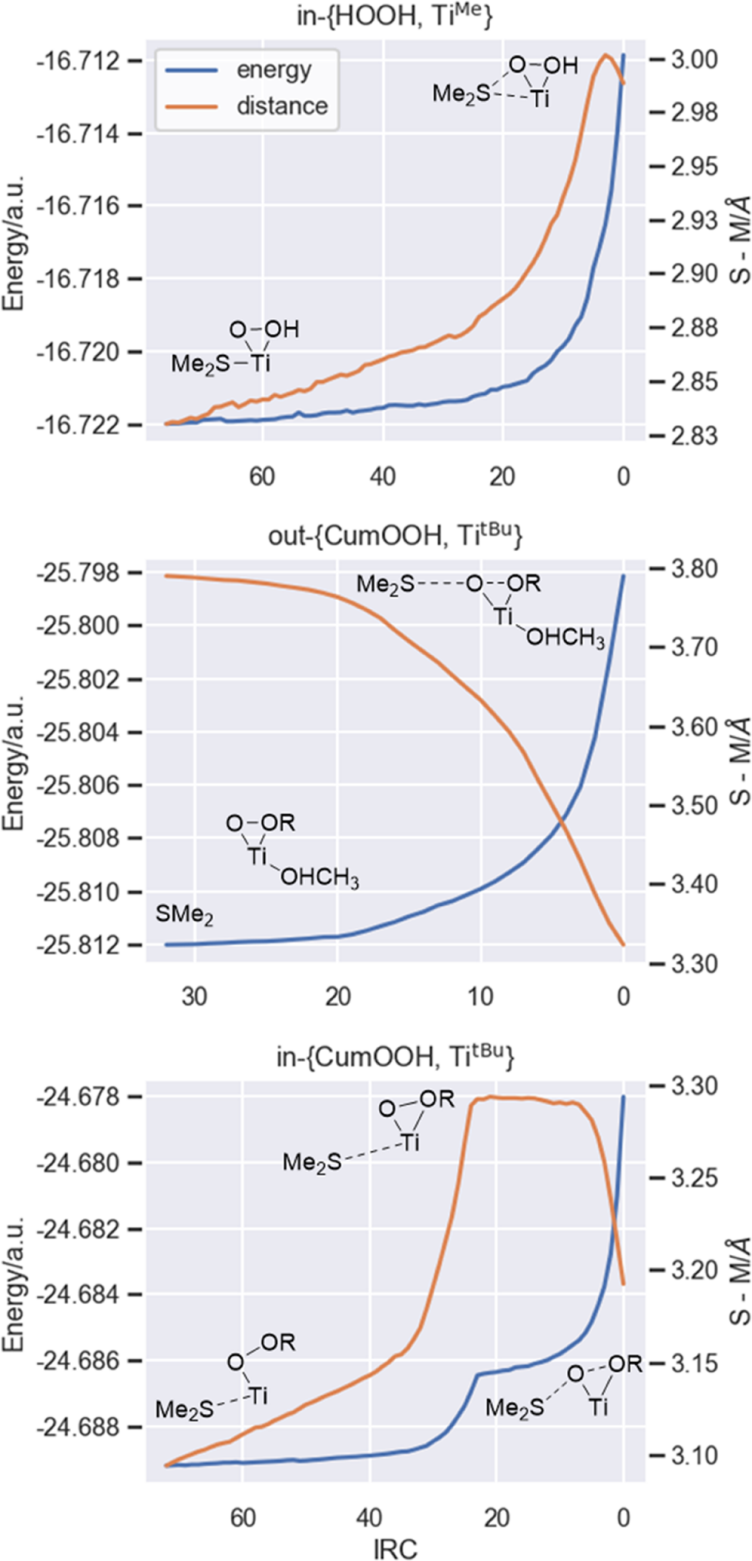
Energy (blue) and the S–M distance (orange) along the reaction coordinate (sulfide–peroxo complex to TS). Chemical structures for the most relevant parts of the curve are also presented.

As stated in the Computational Details section, we have characterized the minima connected by a given transition state by optimizing the structures obtained after small displacements along the reactive normal mode. To improve our understanding of the true attack mode of the sulfide, we also carried out intrinsic reaction coordinate (IRC) calculations on a subset of our titanium TSs: **in-{HOOH,Ti**^**Me**^**}**, **in-{CumOOH,Ti**^**Me**^**}**, **in-{CumOOH,Ti**^**tBu**^**}**, and **out-{CumOOH,Ti**^**tBu**^**}**, shown in Figures 3 and S1.

All IRC curves in Figure 3 will be discussed from the right (**TS3**) to the left (**Int4**), as this is the direction in which the calculation proceeds from the input transition state structure. The **in-{HOOH,Ti**^**Me**^**}** system follows a traditional inner-sphere IRC curve, with a smooth decrease of the S–M distance as we move from the TS (right side of the plot) to the bonded **Int4** structure. **out-{CumOOH,Ti**^**tBu**^**}** exemplifies the outer-sphere behavior, with sulfide getting away from the metal center until the distance is too long (3.80 Å) and no interaction between the moieties remains whatsoever. The profile for **in-{CumOOH,Ti**^**tBu**^**}** shows, instead, a much more complex mixed behavior. During the first steps of the calculation, the sulfide *gets away* from **Ti**, in an outer-like fashion, reaching a quite flat plateau where SMe_2_ is still quite close to the metal (3.3 Å).

The calculation was restarted after IRC step 20, with the side effect of the IRC step size being slightly altered. From there, there is a dramatic shift in the regime: energy drops down and the sulfide quickly approaches the metal until convergence is reached. Simultaneous to the sulfide approach, alkylperoxo reorganization is observed, going from bidentate (as in the TS) to monodentate, as in **Int4**.

The curve for **in-{CumOOH,Ti**^**Me**^**}** (Figure S1 of the Supporting Information) hints at the same behavior: starts outer-like, with sulfide separating from the metal until 3.46 Å, and at the very last step both the energy and S–Ti distance drop abruptly, with the calculation abnormally finishing as if it was converged. Geometry optimization of structures in the first energy/distance plateaus of either **in-{CumOOH,Ti**^**Me**^**}** or **in in-{CumOOH,Ti**^**tBu**^**}** also shows the same sulfide approach/alkylperoxo reorganization observed in the IRC curves.

The rotation and coordination mode switch of the peroxo group is, consequently, a key aspect of the “in” transition states bearing bulky complexes. In fact, the “in” mode seems to occur through a *mixed* two-step process from the sulfide-bonded intermediate **Int4**: (i) Sulfide is partially separated and the peroxide rotates from the η^1^ to η^2^ binding mode and (ii) the S–O bond is formed in an outer-sphere-like fashion.

The association complex between the separated sulfide and the η^2^ peroxo species formed after the first step does not seem to be a true minimum but a *metastable* structure. Both IRC curves and optimization attempts show an uphill slope between **Int4** (η^1^-peroxo and S–Ti bond) and **in-TS3** (η^2^-peroxo and no bonding between S and Ti), without any local barrier for alkylperoxo rotation. In summary, we have that the “in” route presents an inner-like minimum and an outer-like transition state.

The main question left is the nature of the sulfur–metal interaction. This must be strong enough to make the “in” mechanism more feasible than a traditional outer-sphere approach for titanium but also subtle enough to justify the long M–S distances and the partial decoordination upon reaction. As mentioned before, for the reference **{CumOOH, Ti**^**Me**^**}** system, **Int4** has a *covalent* Ti–S interaction according to the Bader analysis, despite the length of the 2.81 Å bond, but **in-TS3** does not. Indeed, not even the most inner-sphere-like **in-{HOOH, Ti**^**Me**^**}-TS3** (S–Ti: 2.98 Å) shows any BCP in between the two atoms. Therefore, the interaction in the TS must be mostly noncovalent. To analyze this, we considered two different approaches: noncovalent interaction^33^ (NCI) and density overlap region interaction^34^ (DORI) analyses. Both techniques provide similar information: a *qualitative* assessment of noncovalent interactions. The major difference between their outputs is that NCI isolates *only* the low-density regions that are purely noncovalent, while DORI shows covalent and noncovalent interactions altogether.

**Figure 4:**
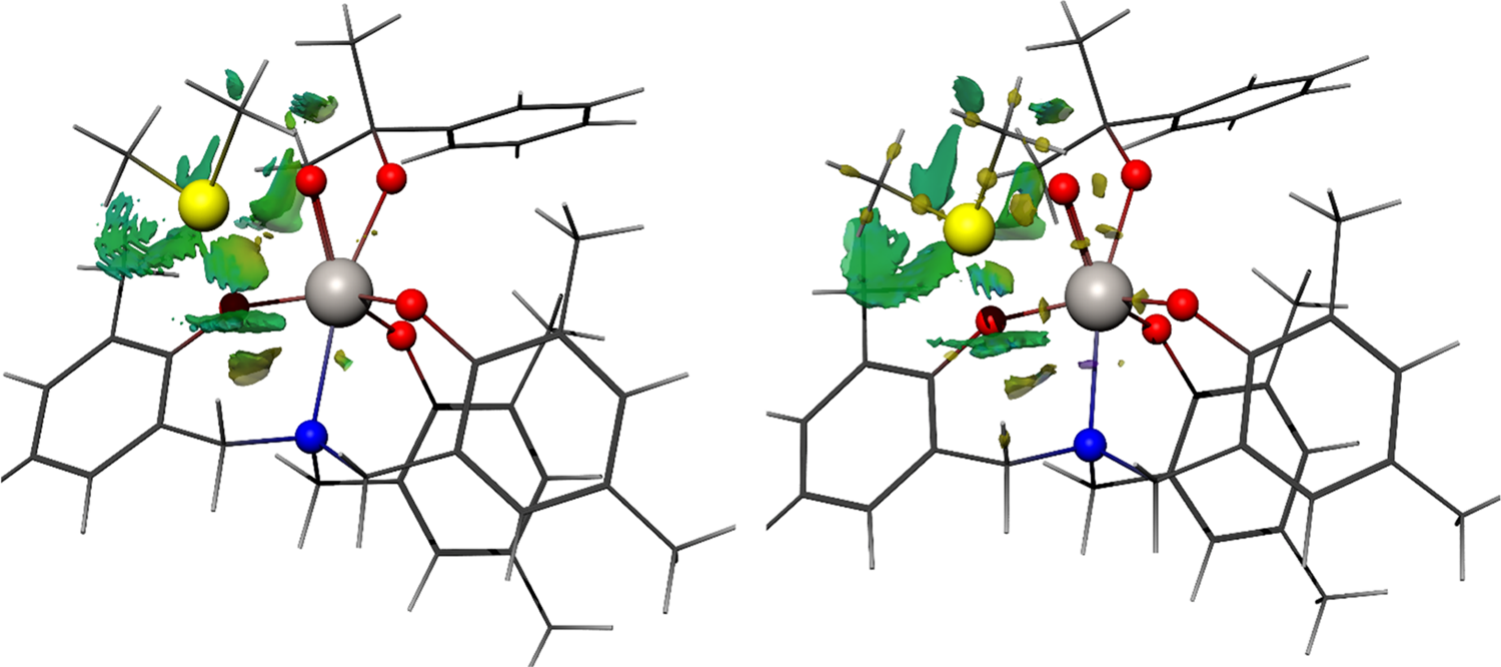
Tridimensional surface plots for the noncovalent interaction (NCI, left, isosurface value= 0.1) and density overlap region interaction (DORI, right, isosurface = 0.99) descriptors for the Ti-in-Cm,Me complex. To focus on the interactions most relevant to the sulfide, only the region within 6.5 angstrom of the sulfur atom is represented. Surface coloring was done through the signed charge density (DenSigned) for both representations, with yellow zones in the DORI isosurface corresponding to the covalent bond and green zones to weaker noncovalent interactions, matching the NCI.

Isosurface representations of the NCI and DORI analyses for **in-{CumOOH,Ti**^**Me**^**}** are shown in Figure 4 for reference, while the corresponding images for the other TS3 variants are collected in the Supporting Information. While the interpretation of these descriptors in such a complex system is not obvious, the main message arising from these representations is that the sulfide–metal complex interaction does not come from a direct S–M linkage. There are important contributions of the weak covalent interaction between S and O, which is highlighted by the DORI analysis, and also from the heteroatoms of the aminotriphenolate ligand. In general, NCI surfaces show that for the “in” structures, sulfur interacts mostly with one O of the ligand and with N, while in the “out” TSs, two O atoms from the ligand are involved instead. Additionally, steric clashes between alkyl and aryl moieties, and also π-stacking between aryl rings also contribute. All in all, and in line with the whole previous discussion, the interaction between the two subunits in the transition state cannot be ascribed to a well-defined bond but instead to a complex network of interactions coming from the substrate, ligand, and metal.

**Figure 5:**
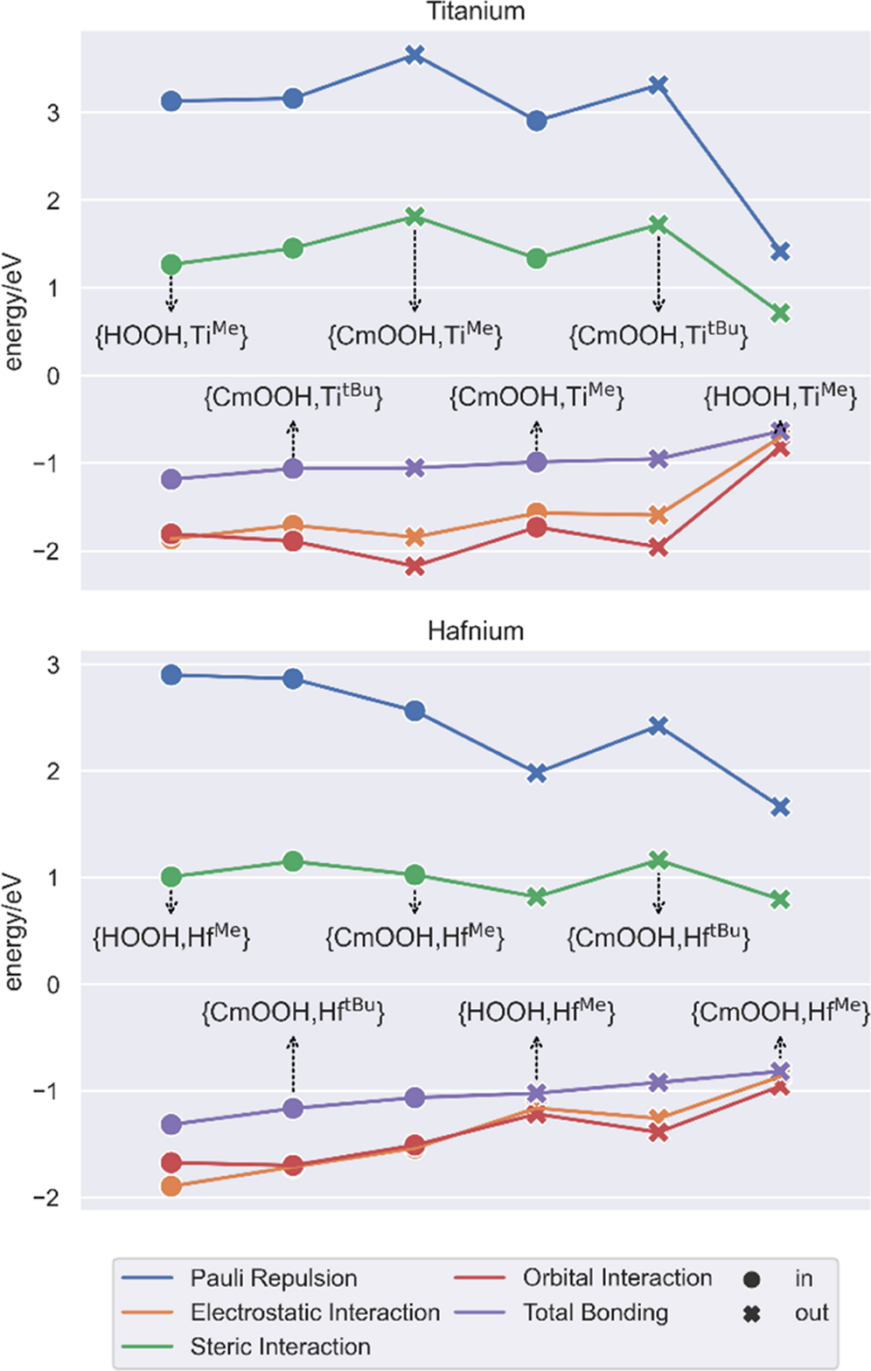
Fragment-based energy decomposition for TS3 variants.

To complete the description, we carried out a bonding energy decomposition analysis (BDE)^26^ on the 12 TS3 structures, with the SMe_2_ group as one fragment and the metal complex as the other. In this way, we can get a more quantitative assessment of the intricate sulfide/metal bonding nature. As shown in Figure 5, all sulfide/complex bonding energies are negative, accounting for a favorable interaction overall. In the case of titanium, the **HOOH, Ti**^**Me**^ transition states are, as expected by their geometries (Figure 2), the closest to the limit pure-inner- and pure-outer-sphere situations. The “in” structure shows strong repulsive (Pauli repulsion and steric) and attractive (orbital and electrostatic) interactions, hinting at a more traditional bond. In contrast, the corresponding “out” transition state has much milder interactions on both sides: the two fragments do not interact as much. When the bulkier **CumOOH, Ti**^**Me**^, and **CumOOH, Ti**^**tBu**^ systems are taken into account the in/out differences are, yet again, blurred, due to the mixed character of the “in” route. In this case, the “out” structures have more repulsion and orbital interactions than the “in” analogues.

For the case of hafnium, the general behavior is more straightforward, like in the distance/angle mapping (Figure 2), and all “out” transition states have weaker sulfide/metal bonding than their “in” counterparts. Nevertheless, despite being less anomalous than its smaller group partner, sulfide activation in Hf still cannot be properly described as completely inner- or outer-sphere and is also clearly affected by the bulk of the substrate and the substituents carried out by the ligand.

## Conclusions

The computational characterization of the catalytic cycles for the **Ti**- and **Hf**-aminotriphenolate-catalyzed sulfoxidation process is in good agreement with the experimental evidence. The proposed mechanism explains the remarkably faster kinetics of the Hf complex and its preference for the sulfone product (second oxidation), compared to **Ti** that produces a larger ratio of sulfoxide (first oxidation).

Furthermore, the thorough analysis of the nature of the sulfoxidation transition state sheds new light on the long-asked question of substrate activation in peroxide-based oxidation reactions. For the case of **Ti**, the most favorable route for the first oxidation step is clearly the “in” pathway, which is a mixed inner/outer mechanism: starting from a sulfide-bonded intermediate, whose corresponding transition state is more outer-like. For **Hf**, the in/out energy differences are less accentuated and the “out” routes are only slightly more favored, with the geometry of the TS still being very sensitive to changes in the substitution pattern of the catalyst or the peroxide. Our findings show how the traditional categories of inner and outer-sphere attacks fall short when dealing with realistic, complex catalytic systems. We propose that there is a whole spectrum of possible substrate activation processes that intertwine characteristics of the traditional inner- and outer-labels. The exact placing of a given structure along this spectrum depends on many factors, such as the size of the metal center, the bulkiness of the entering peroxide, or the substitutions in the substrate and ligand scaffold.

Finally, through the combination of Bader, NCI, DORI, and BDE analyses on different variants of the “in” and “out” transition states proposed in this work, we have shown how the general interaction between the sulfide and the catalyst eventually depends on an intricate network of weak interactions. All in all, the complexity of the overall picture explains why such a seemingly simple question as sulfide binding to peroxo complexes has been so discussed over the years: the traditional pathways (Scheme 1) are intrinsically blended and modulated by electronic and steric factors.

## Supporting Information Available

Synthetic procedure, characterization data, and calculations (PDF)

## Notes

All DFT calculations are available as a dataset collection in the ioChem-BD repository^35^ and can be accessed at https://doi.org/10.19061/iochem-bd-1-225.
