# Supplementary material for: Elucidating Sulfide Activation Mode in Metal-Catalyzed Sulfoxidation Reactivity
Source: Inorg Chem. 2022 Feb 28;61(10):4494–501. doi: 10.1021/acs.inorgchem.2c00037 (PMC8924929; doi:10.1021/acs.inorgchem.2c00037)
Supplement: Supplementary file 1 — ic2c00037_si_001.pdf [file ic2c00037_si_001.pdf]

Supporting Information for

Elucidating Sulfide Activation Mode in Metal Catalyzed Sulfoxidation Reactivity

Authors: Diego Garay-Ruiz,‡, § Cristiano Zonta,#,* Silvia Lovat,# Joan González-Fabra,‡ Carles Bo‡,§,* and Giulia Licini‡,*

a Department of Chemical Sciences, University of Padova, via Marzolo 1, 35131 Padova

*Correspondence to: cristiano.zonta@unipd.it, giulia.licini@unipd.it, cbo@iciq.cat

Contenido

Supporting Information for 1
1 General Methods 2
2 Synthesis and Characterization 3
2.1 Synthesis of Hf 3
3 Calculations 4
3.1 Ligand bonding on M-hydroperoxo complexes 4
3.2 Energies for in- and out- sulfoxidation transition states. 4
3.3 IRC calculation for Ti-in-Cm,Me 5
3.4 NCI and DORI representations 6
3.5 Bader analysis 9

1 General Methods

NMR spectra were recorded at 301 K on Bruker 400 Avance III BBi-z grad 5 mm and Bruker Avance-III 500MHz instruments. All the ^{1}H-NMR spectra were referenced to residual isotopic impurity of the solvent. The following abbreviations are used in reporting the multiplicity for NMR resonances; s=single, d=doublet, t= triplet, and m=multiplet. The NMR data were processed using Bruker Topspin 3.5 pl2 and MestReNova 12.0.0. Chemicals were purchased from Merck, TCI, or Apollo Scientific and used without further purification.

2 Synthesis and Characterization

Synthesis of the ligand and Ti complex have been reported in Dalton Trans., **2010**,39, 7384-7392

2.1 Synthesis of Hf

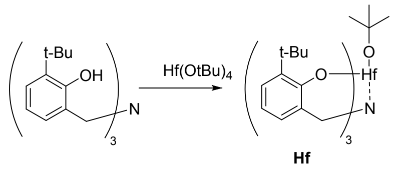

Complex **Hf** was prepared in glovebox by mixing homogeneous solutions of the corrisponding ligand (0.0495 M) and $Hf(Ot\text{-}Bu)_4$ (0.0495 M) in $CDCl_3$ in a 1:1 ratio to a final concentration 0.0124 M of the complex. The solution was used for kinetic experiments without further purifications and without removing the three equivalents of t-BuOH released from the metal precursor.

^{1}H-NMR (250 MHz, $CDCl_3$): d 7.24 (d, 3H, J = 7.8 Hz, ArH), 6.95 (d, 3H, J = 7.7 Hz, ArH), 6.69 (t, 3H, J = 7.8 Hz, ArH), 3.51 (s, 6H, NCH_2), 1.48 (9H, s, C $(CH_3)_3$), 1.46 (s, 27H, t-Bu).

ESI-MS: 721.2 (M-O-t-bu+OH+Na). In the NMR spectra resonances relative to free tert-butanol released in the reaction are present: $_1$H-NMR (250 MHz, $CDCl_3$): δ 1.24 (9H, s, $C(CH_3)_3$).

3 Calculations

3.1 Ligand bonding on M-hydroperoxo complexes

As addressed in the main text, the expansion of the coordination sphere of the M-peroxo complex **Int3** from a bipyramidal-like geometry to an octahedral-like disposition is one of the key aspects of the overall reaction mechanism. For simplicity, we will be considering the hydroperoxo complexes derived from the **{H, Me}** model, to decouple the coordination from the observed denticity changes observed for the alkylperoxo groups, where the bulky cumyl group hinders the η^2 binding mode.

Table S1. Gibbs free energies, in kcal·mol^{-1}, for different equatorial ligands on the η^2-M-OOH complexes derived from Int3.

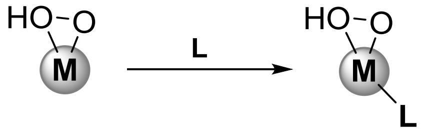

ΔG	Ti	Hf
- SMe_2	5.3	-1.1
- $O{=}SMe_2$	1.4	-8.2
- $S(O)Me_2$	10.6	4.2
- $O(H)CH_3$	2.0	-4.8
- $ONMe_3$	-8.6	-15.0

There are large differences in the binding strength of the different ligands on the model system, as depicted in Table S1. In line with our expectations, Hf is more prone to add additional ligands than Ti, and consequently the ΔG values for the ligand binding are mostly *negative* for Hf and *positive* for Ti. For the two metals, the order of increasing ligand strength is:
S-bonded sulfoxide << sulfide < methanol < O-bonded sulfoxide << trimethyl N-oxide.

This confirms the relevance of the product/catalyst interplay, as sulfoxide attains more stable complexes than methanol (when it reacts through O), and thus will displace the alcohol from the metal center, accessing the reaction channels proposed in the main text. On the other hand, the very strong Lewis base trimethyl N-oxide forms even more stable structures and would then displace any other reactant in the medium, substantially lowering the resting state of the system and slowing down the reaction.

3.2 Energies for in- and out- sulfoxidation transition states.

Table S2. Relative potential and Gibbs free energies, in kcal·mol^{-1}, for the in- and out- attack modes for the two oxidation stages with Ti and Hf catalysts.

	Complex	E/kcal·mol^{-1}	G/kcal·mol^{-1}
Ti	Ti-TS3-1ox-in	-0.5	*9.0*
	Ti-TS3-2ox-in (via S)	0.3	11.1
	Ti-TS3-1ox-out	-4.3	17.9
	Ti-TS3-2ox-out	-5.7	17.5
Hf	Hf-TS3-1ox-in	-6.0	3.0
	Hf-TS3-2ox-in	-10.3	1.3
	Hf-TS3-1ox-out	-17.2	0.9
	Hf-TS3-2ox-out	-20.8	*-1.6*

All energy values in Table S2 are referred to the reference state M-OR + **S/SO** + ROOH. As mentioned in the main text, it is shown how Ti strongly disfavors the outer-sphere mechanisms, much higher in energy both for the first and the second oxidation processes. In contrast, the differences for Hf are much more subtle, with the out transition states being more favorable by only 2.1 (1ox) and 2.9 kcal·mol^{-1} (2ox) compared to the in analogues.

3.3 IRC calculation for Ti-in-Cm,Me

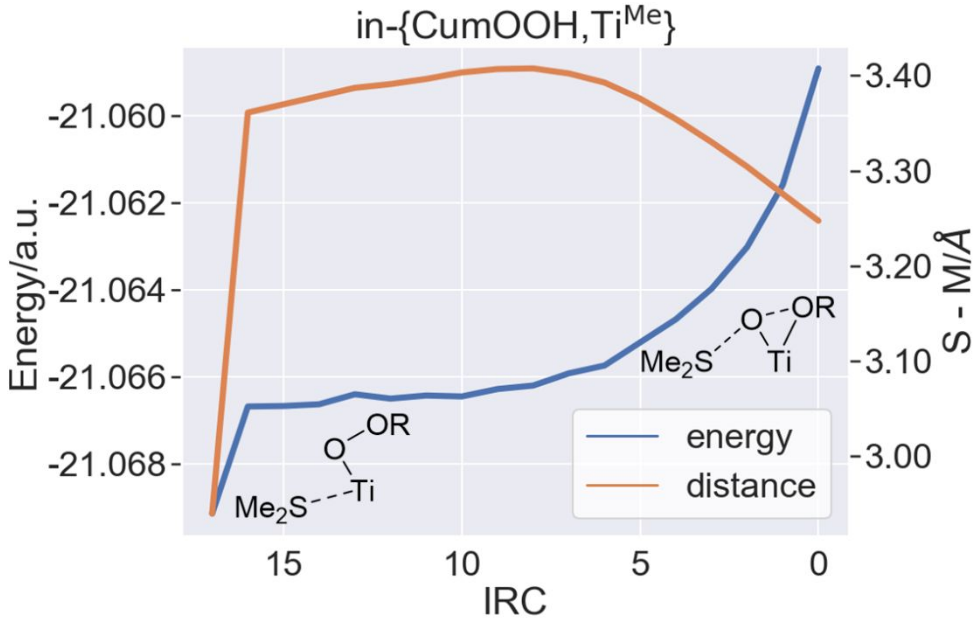

Figure S1. Energy (blue) and S - M distance (orange) along the IRC from TS3-Ti-in-{Cm,Me}.

Figure S1 shows the mixed inner/outer behavior of the "in" routes, as the analogous plot for **Ti-in-{Cm,tBu}** presented in the manuscript. It starts outer-like, with sulfide separating from the metal until 3.46 Å, and at the very last step both energy and S–Ti distance drop abruptly and the calculation terminates abnormally, like if it was converged. Therefore, in a single IRC step the S–Ti distance decreases more than 0.4 Å, suddenly switching from a non-bonded to a clearly bonded state. This unexpected termination serves as another confirmation of the complexity of the sulfur/metal interaction and the mixed character of the sulfoxidation transition states.

3.4 NCI and DORI representations

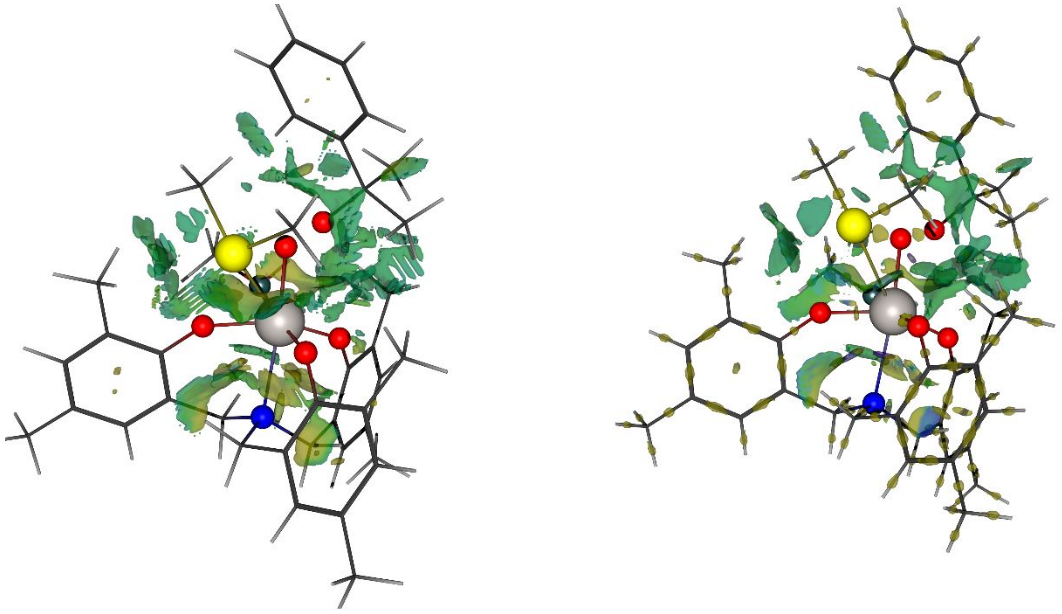

Figure S2. NCI (left, isosurface = 0.1) and DORI (right, isosurface = 0.99) representations for Ti-out-Cm,Me. Surface is colored according to the signed charge density (DenSigned).

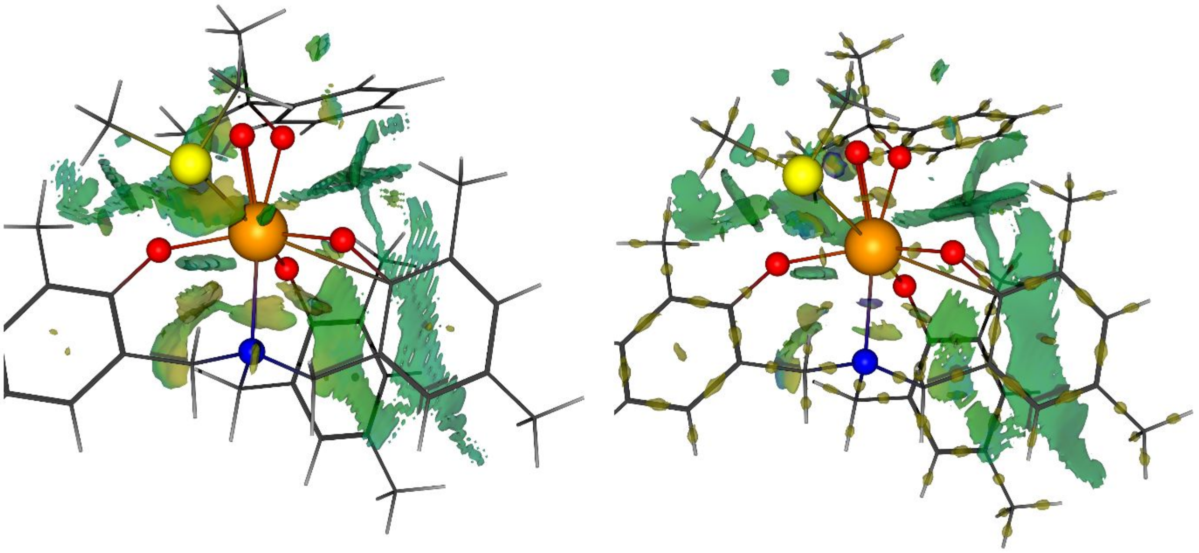

Figure S3. NCI (left, isosurface = 0.1) and DORI (right, isosurface = 0.99) representations for Hf-in-Cm,Me. Surface is colored according to the signed charge density (DenSigned).

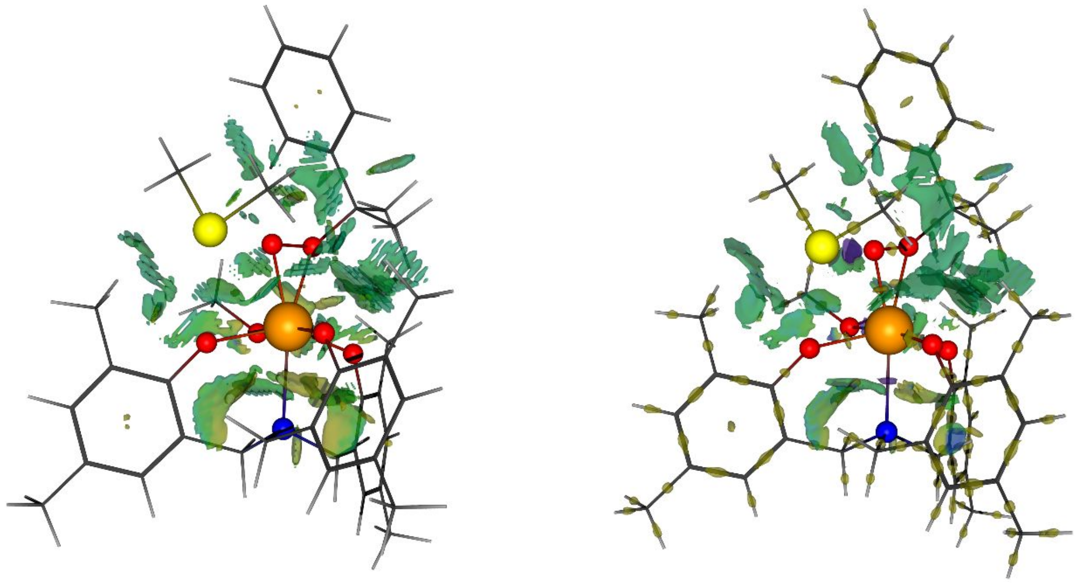

Figure S4. NCI (left, isosurface = 0.1) and DORI (right, isosurface = 0.99) representations for Hf-out-Cm,Me. Surface is colored according to the signed charge density (DenSigned).

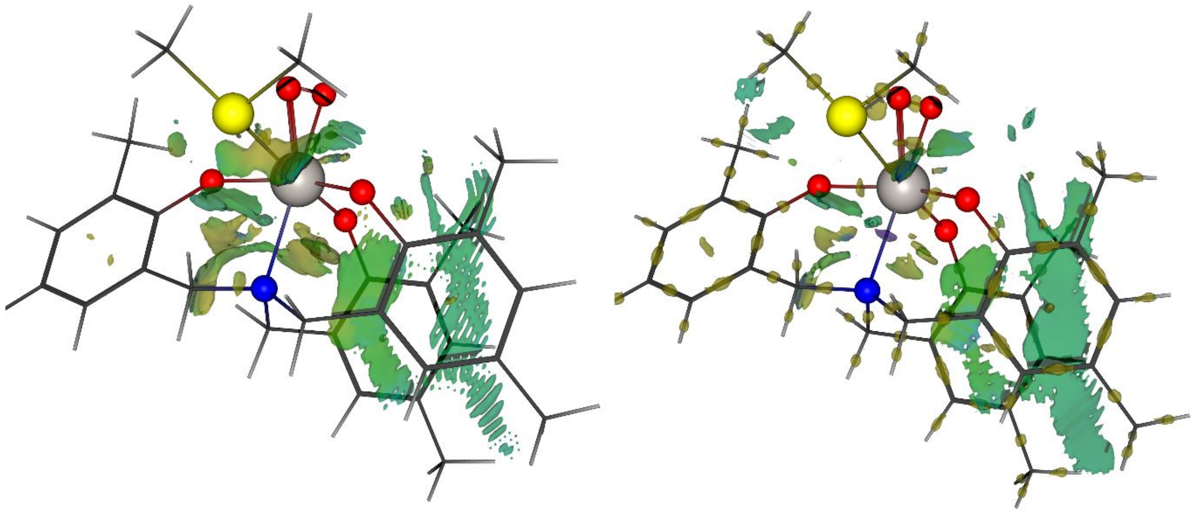

Figure S5. NCI (left, isosurface = 0.1) and DORI (right, isosurface = 0.99) representations for Ti-in-H,Me. Surface is colored according to the signed charge density (DenSigned).

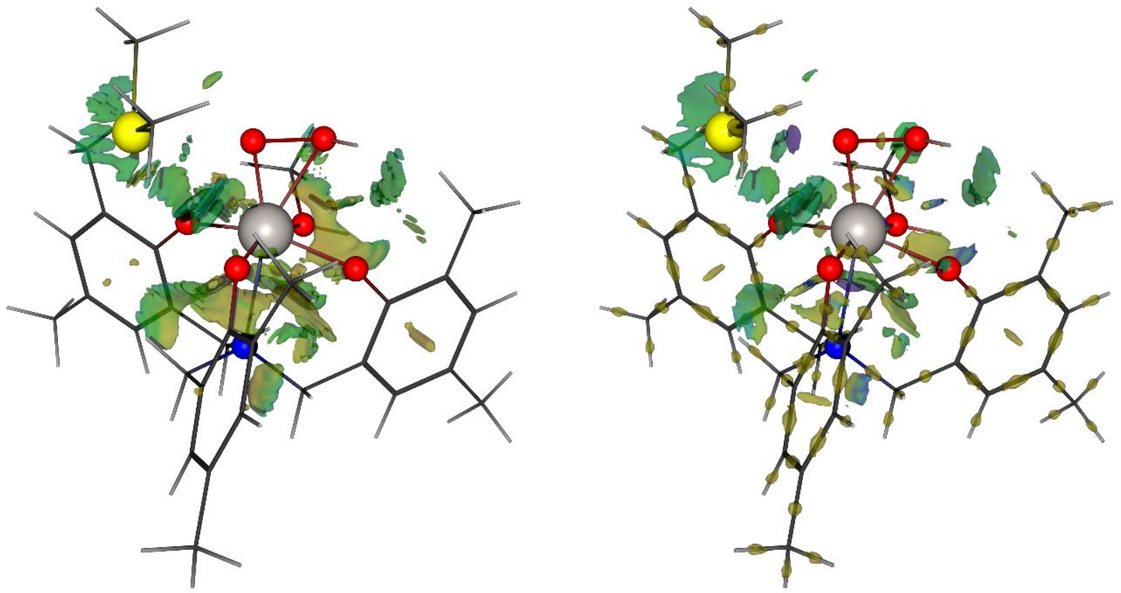

Figure S6. NCI (left, isosurface = 0.1) and DORI (right, isosurface = 0.99) representations for Ti-out-H,Me. Surface is colored according to the signed charge density (DenSigned).

Figure S2 - Figure S6 depict the diversity of sulfide/metal complex interactions across some of the most relevant variants of the sulfoxidation transition state **TS3**. Direct S–M non-covalent interactions appear only for **Ti-in-{H,Me}** and **Hf-in-{Cm,Me}**, but additional interactions between S and the heteroatoms in the ligand scaffold contribute to hold the sulfide into place. Moreover, DORI reveals a weak covalent interaction between S and the O in the peroxo group common to all structures.

3.5 Bader analysis

Bader's Atom In Molecules (AIM) analyses were carried out to detect covalent interactions in some of the key structures of the mechanism for Ti: the sulfur-bonded intermediate **Int4** and the **in** and **out** variants of **TS3**. In all plots, *orange* spheres correspond to bond critical points, which mark a covalent interaction between two basins. *Green* spheres represent ring critical points and *cyan* ones, cage critical points.

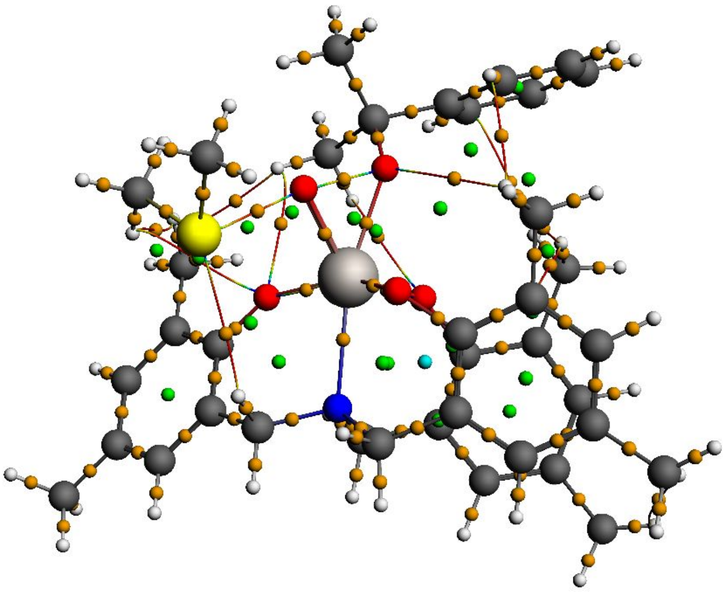

Figure S7. Atoms in Molecules (AIM) analysis for Ti-ivn-TS3-Cm,Me.

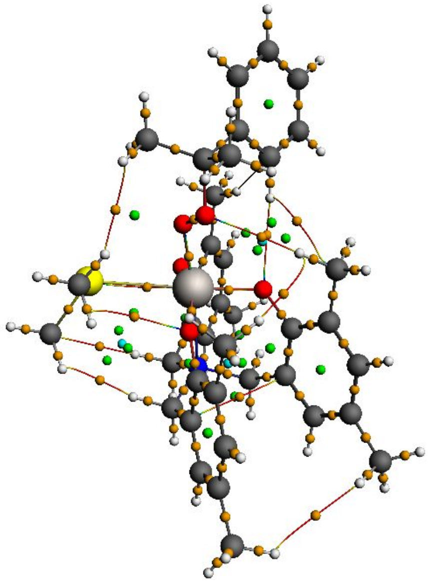

Figure S8. Atoms in Molecules (AIM) analysis for Ti-Int4-Cm,Me.

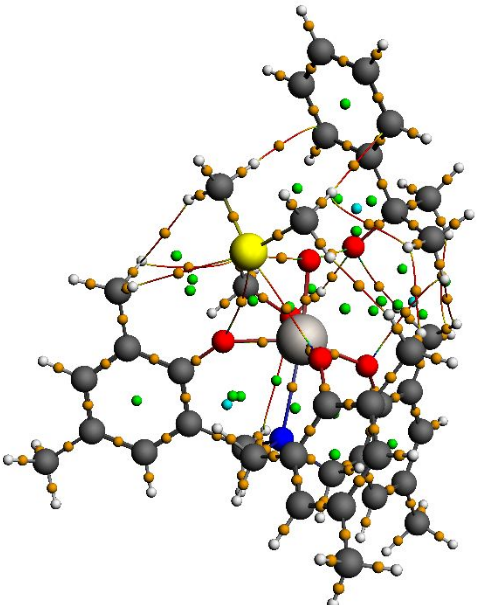

Figure S9. Atoms in Molecules (AIM) analysis for Ti-out-TS3-Cm,Me.

In general, AIM highlights complex interactions for all structures. **Int4** (Figure S8) shows a covalent bond between Ti and S, which is not present in the transition states. The interactions with the ligand O atoms for **out-TS3-Cm,Me** appearing in the NCI and DORI analyses in Figure S2 have a non-negligible covalent character, as shown by the presence of BCPs in Figure S9. In contrast, the interactions for **in-TS3-Cm,Me** are only non-covalent, without any sulfur – heteroatom BCP in Figure S7 apart from the interaction with the peroxo group.
